# A Mobile App for Identifying Individuals With Undiagnosed Diabetes and Prediabetes and for Promoting Behavior Change: 2-Year Prospective Study

**DOI:** 10.2196/10662

**Published:** 2018-05-24

**Authors:** Angela YM Leung, Xin Yi Xu, Pui Hing Chau, Yee Tak Esther Yu, Mike KT Cheung, Carlos KH Wong, Daniel YT Fong, Janet YH Wong, Cindy LK Lam

**Affiliations:** ^1^ Centre for Gerontological Nursing, School of Nursing The Hong Kong Polytechnic University Hong Kong SAR China (Hong Kong); ^2^ School of Nursing The University of Hong Kong Hong Kong China (Hong Kong); ^3^ Department of Family Medicine and Primary Care The University of Hong Kong Hong Kong China (Hong Kong); ^4^ Centre on Research and Advocacy The Hong Kong Society for Rehabilitation Hong Kong China (Hong Kong)

**Keywords:** diabetes mellitus, prediabetes, prediabetic state, mobile apps, lifestyle

## Abstract

**Background:**

To decrease the burden of diabetes in society, early screening of undiagnosed diabetes and prediabetes is needed. Integrating a diabetes risk score into a mobile app would provide a useful platform to enable people to self-assess their risk of diabetes with ease.

**Objective:**

The objectives of this study were to (1) assess the profile of Diabetes Risk Score mobile app users, (2) determine the optimal cutoff value of the Finnish Diabetes Risk Score to identify undiagnosed diabetes and prediabetes in the Chinese population, (3) estimate users’ chance of developing diabetes within 2 years of using the app, and (4) investigate high-risk app users’ lifestyle behavior changes after ascertaining their risk level from the app.

**Methods:**

We conducted this 2-phase study among adults via mobile app and online survey from August 2014 to December 2016. Phase 1 adopted a cross-sectional design, with a descriptive analysis of the app users’ profile. We used a Cohen kappa score to show the agreement between the risk level (as shown in the app) and glycated hemoglobin test results. We used sensitivity, specificity, and area under the curve to determine the optimal cutoff value of the diabetes risk score in this population. Phase 2 was a prospective cohort study. We used a logistic regression model to estimate the chance of developing diabetes after using the app. Paired *t* tests compared high-risk app users’ lifestyle changes.

**Results:**

A total of 13,289 people used the app in phase 1a. After data cleaning, we considered 4549 of these as valid data. Most users were male, and 1811 (39.81%) had tertiary education or above. Among them, 188 (10.4%) users agreed to attend the health assessment in phase 1b. We recommend the optimal value of the diabetes risk score for identifying persons with undiagnosed diabetes and prediabetes to be 9, with an area under the receiver operating characteristic curve of 0.67 (95% CI 0.60-0.74), sensitivity of 0.70 (95% CI 0.58-0.80), and specificity of 0.57 (95% CI 0.47-0.66). At the 2-year follow-up, people in the high-risk group had a higher chance of developing diabetes (odds ratio 4.59, *P*=.048) than the low-risk group. The high-risk app users improved their daily intake of vegetables (baseline: mean 0.76, SD 0.43; follow-up: mean 0.93, SD 0.26; *t*_81_=–3.77, *P*<.001) and daily exercise (baseline: mean 0.40, SD 0.49; follow-up: mean 0.54, SD 0.50; *t*_81_=–2.08, *P*=.04).

**Conclusions:**

The Diabetes Risk Score app has been shown to be a feasible and reliable tool to identify persons with undiagnosed diabetes and prediabetes and to predict diabetes incidence in 2 years. The app can also encourage high-risk people to modify dietary habits and reduce sedentary lifestyle.

## Introduction

Prevention of diabetes is at the top of the agenda for health promotion worldwide and in Hong Kong [1-3]. Target populations for diabetes prevention include those who have not had a diagnosis of diabetes and those who are in the stage of prediabetes [4]. Early detection of individuals with undiagnosed diabetes and prediabetes (UDPD) would enhance the implementation of lifestyle modification interventions, which have been shown to prevent the progression to diabetes or further complications [5]. It is estimated that 193 million people, or nearly half of those with diabetes, around the world have undiagnosed diabetes [6].

mHealth has been used for different purposes in health promotion and maintenance. The proliferation of mobile phones and software apps has provided a new channel for health promotion, including symptom recording [7], smoking cessation [8], and weight control [7]. The advantage and convenience of the compact size and mobility of mobile phones allows users to access health information and health assessment tools at any time and at any place that best suit individuals’ pace of living. A research study found that 75 million adults in the United States used their mobile phones for health information and as tools [9]. Among those aged 55 years and older who owned mobile phones or tablets, half used the devices for health purposes [9]. The use of mobile apps has been evolving and becoming popular in Chinese society as mHealth is considered as not only trendy but also practical and layman friendly. In Hong Kong, 12 free-of-charge health and fitness apps in the Android app market have been labelled as the most popular apps, and each app has had more than 250,000 downloads as of 2011 [10]. However, scarce evidence about mHealth in assessing diabetes risk has been documented in the Chinese population.

Integration of a diabetes risk score into a mobile app is an innovative and potentially powerful tool for promoting diabetes self-assessment. Evidence has shown that the Finnish Diabetes Risk Score (FINDRISC) has supported diabetes screening and prevention because of its cheap, easy-to-administer, convenient, and noninvasive features [11]. Laypeople can easily use FINDRISC to assess their risk of developing diabetes, without any training. It is now being used in various European countries, including Finland, Belgium, Sweden, Greece, Germany, and Spain [12-17]. Because of the popularity, reliability, and user-friendly nature of FINDRISC, we chose it as the key measurement of the Diabetes Risk Score (DRS) mobile app.

The DRS app was developed by a university project team comprising a nursing faculty with rich experience in health literacy interventions, 2 family medicine experts, a professor in endocrinology, and a statistician. User tests were conducted with 22 Chinese adults in April 2013. Half of the participants of the user test had secondary education or above. Comments were collected from these app users on the clarity of the instructions given in the app, the logic of the sequence of the questions in the app, and the ease of data input by the participants. The app was then revised according to their comments. The DRS app (version 2) was officially launched on August 28, 2014 in a media interview with 11 newspapers and 1 electronic media, who reported its launch. The Quick Response code of the app was posted on the university website to promote the app. This free-of-charge app could be downloaded from both the Google Store (for Android devices) and the App Store (for iOS devices; eg, iPad, iPhone) by searching the term “HKUDRS.”

The DRS app included the Chinese FINDRISC and questions related to lifestyle (such as smoking, drinking, dietary pattern, and physical activity engagement). In the DRS app, the exact diabetes risk score was not shown, but the risk level was shown in a figure in which a pointer fell in one of the two color zones, with red indicating high risk and green indicating low risk.

The objectives of this study were to (1) assess the profile of DRS mobile app users, (2) determine the optimal cutoff value of the diabetes risk score to identify UDPD in the Chinese population, (3) estimate users’ chance of developing diabetes within 2 years of using the app, and (4) investigate high-risk app users’ lifestyle behavior changes after ascertaining their risk level from the app.

## Methods

### The 2-Phase Study

We divided the whole study into 2 phases. We conducted phase 1 from August 2014 to October 2016, using a cross-sectional design. Phase 1a assessed the users’ profile, while phase 1b assessed the appropriate cutoff value of the diabetes risk score to identify UDPD in the Chinese population. We conducted phase 2 from October 3, 2016 to November 6, 2016 with a prospective cohort design. Phase 2a followed up the app users to estimate their chance of developing diabetes within 2 years. Phase 2b assessed the app users’ lifestyle changes after knowing their risk of diabetes from the app.

### Samples

Since this is a free app in the app stores, anyone who was capable of accessing the internet and app stores and of reading and understanding Chinese could download the app to their mobile phones. On the first screen of the app, we indicated that it was developed for research purposes, and its use implied that users agreed to join the research study and consented to having the project team use their data in aggregate for research purposes and future analysis. We included those who met the following criteria in the analysis in phase 1a: (1) aged 18 years or over, and (2) having a phone number indicating the country code 852 (for Hong Kong). We selected participants for phase 1b from phase 1a who met the following criteria: (1) provided phone or email addresses in the app and agreed to allow the project team to approach them, (2) had never had a diagnosis of diabetes (of any kind), and (3) were willing to attend a comprehensive health assessment (including blood taking) in a university campus. Participants in phase 2 were those who (1) used the DRS app in 2014 and 2015, (2) provided email addresses in the app, and (3) were willing to complete an online survey. We excluded those with known diabetes in 2014 and 2015 or those who used the app less than 1 year from the time we conducted the online survey.

### Sample Size Calculation

We calculated the sample size for phase 1b for the receiver operating characteristic (ROC) curve analysis using MedCalc software version 15.8 (MedCalc Software bvba). We assumed that an area under the curve (AUC) of 0.70 for a particular test was significant from the null hypothesis value of 0.5. The prevalence of people with UDPD in Hong Kong was almost 14% [18,19], and the number of negative cases was approximately 6 times that of positive cases. Assuming the type I error rate was 0.05 and the type II error rate was 0.20, the sample size required for phase 1b was 133.

We calculated the sample size for phase 2a using G*power version 3.1.9.2. With reference to the previous studies, the OR of developing diabetes among persons with UDPD was 4.5 [20], the prevalence of UDPD in Hong Kong was nearly 14% [18,19], and the incidence rate of diabetes among persons with normoglycemia was 0.05 [21]. To achieve a statistical power of 80% and a 5% level of significance to detect the assumed OR, 292 participants were needed.

### Measures

We calculated diabetes risk score by using the FINDRISC formula and the score for the following 8 items in the app: age (<45 years=0, 45-54 years=2, 55-64 years=3, >64 years=4), body mass index (BMI; <25 kg/m^2^=0, 25-30 kg/m^2^=1, >30 kg/m^2^=3), waist circumference (for men: <94 cm=0, 94-102 cm=3, >102 cm=4; for women: <80 cm=0, 80-88 cm=3, >88 cm=4), history of using drugs for high blood pressure (no=0, yes=2), history of being told by health professionals about the possibility of having high blood glucose (no=0, yes=5), participation in physical activity every day (yes=0, no=2), habit of consuming fruit and vegetables every day (yes=0, no=2), and family history of diabetes (no=0, yes=5). We calculated the total risk score by adding the scores of all items, with a possible range from 0 to 26 [15]. App users could key in their body weight (in kilograms or in pounds) and body height (in meters or in inches). The app automatically converted the figures and calculated the BMI using the formula body weight (in kilograms) divided by the square of the height (in meters). App users were also asked to key in their waist circumference to the nearest 0.5 cm.

### Procedures

In phase 1a, users input data into the app on their own, and the data included the 8 items (such as daily intake of fruit and vegetables and daily physical activity) that we used to calculate the users’ diabetes risk. Following the launch of the app, we periodically monitored the number of downloads and ensured that the app was downloadable from the app stores. For the sake of protecting privacy, only the principal investigator (AYML) had right of access to the server. Before passing the data to the trained research assistant for data cleaning and to develop the database for this study, the principal investigator removed all app users’ personal data to protect privacy.

In phase 1b, we sent emails to the app users and invited them to receive a 1-hour comprehensive health assessment in the university campus between June and August 2015. The inclusion criteria were stated clearly in the invitation emails, and the app users replied to the emails and indicated their willingness to join the health assessment. In the assessment, a research nurse took 5 mL of venous blood from each participant. The blood samples were sent to the laboratory of a regional public hospital in which glycated hemoglobin (HbA_1c_) was measured by high-performance liquid chromatography (Variant II Turbo Hemoglobin Testing System, Bio-Rad Laboratories, Inc, Hercules, CA, USA). According to the American Diabetes Association, HbA_1c_ of 48 mmol/mol (6.5%) or greater is considered to indicate diabetes, while HbA_1c_ of 39 mmol/mol (5.7%) or greater but less than 48 mmol/mol (6.5%) is considered to indicate prediabetes [22].

We contacted app users with HbA_1c_ levels higher than 48 mmol/mol (6.5%) by phone and encouraged them to consult their family doctors. We did this for ethical reasons, so that the app users with abnormal readings were not placed at a disadvantage by not receiving necessary treatments.

For phases 2a and 2b, we sent invitation emails to the app users and encouraged them to complete a follow-up questionnaire via Zoho Survey (Zoho Corporation) from October 3, 2016 to November 6, 2016. We identified duplicate inputs by checking the respondents’ email addresses. We deleted a few duplicate inputs before doing the analysis. Diabetes incidence was recorded when the app users self-reported having diabetes during this period. If the app users had a diagnosis of diabetes, we asked them to provide the dates of receiving the diagnosis (month and year). We also asked app users to input their daily intake of fruit and vegetables, daily physical activity, and the relevant diabetes-related items in the questionnaire.

### Ethics Approval and Consent to Participate

We obtained approval from the Institutional Review Board of the University of Hong Kong/Hospital Authority Hong Kong West Cluster. The consent form was embedded on the first screen of the app. App users clicked a button on the app to indicate their consent to join the study.

### Statistical Analyses

We considered duplicate inputs, incomplete inputs, or inputs that were exactly the same as the default figures in blood pressure, body height, and body weight as invalid inputs and excluded these from the analysis. We computed descriptive statistics, including means, standard deviations, frequencies, and percentages, to present the participants’ sociodemographic information and the distribution of diabetes risk scores. In phase 1b, we used ROC curve analysis to determine the optimal cutoff level of diabetes risk scores with reference to the usual clinical practice for suspecting the risk of diabetes (or the stage of prediabetes) in Hong Kong. We also determined the optimal cutoff point of the diabetes risk score by the sensitivity, specificity, maximum value of the Youden index (sensitivity + specificity – 1), and Cohen kappa of the diabetes risk score with reference to the agreement with HbA_1c_. Cohen kappa values of zero or less indicated no agreement, 0.01 to 0.20 none to slight, 0.21 to 0.40 fair, 0.41 to 0.60 moderate, 0.61 to 0.80 substantial, and 0.81 to 1.00 almost perfect agreement [23].

In phase 2a, we used multiple logistic regression models to examine the OR of diabetes incidence between the high-risk group and the low-risk group (classified by the recommended FINDRISC cutoff value found in phase 1b). Educational level is a confounder in diabetes. Many studies have proved that people with a higher educational level have a better lifestyle in their daily lives, and people with a healthy lifestyle will have less risk of developing diabetes in the future [24]. A meta-analysis also demonstrated that higher educational levels were consistently associated with lower incidence of diabetes [25]. As sex and education were correlated, we adjusted both sex and education in the regression models. We used the Hosmer-Lemeshow test to assess the goodness of fit for the logistic regression models [26]. The model fits the data well when the *P* value is greater than .05 [26]; this implies the model has acceptable fitness.

In phase 2b, we applied paired *t* tests to compare the means of lifestyle variables at the baseline with those at follow-up for the high-risk group. We analyzed the data using IBM SPSS version 22.0 for Windows (IBM Corporation).

## Results

### Phase 1a: Profile of Diabetes Risk Score App Users

We collected data in the period of August 28, 2014 to December 31, 2016. A total of 13,289 Chinese residents downloaded the DRS app and self-assessed their risk. After cleaning the data, we considered 4549 as valid data in phase 1a. A total of 3171 (69.71%) were Android users and the rest were iPhone users. The mean (SD) diabetes risk score was 9.10 (SD 4.85). A total of 1042 (22.91%) were shown to be at risk of developing diabetes. Table 1 shows the demographics of the app users in all phases. Most app users (2738/4549, 60.19%) were male; 1328 (29.19%) were aged 55 to 64 years and 1606 (35.30%) were aged 65 years or above. Nearly two-fifths of the app users had tertiary education or above (1911/4549, 42.01%).

### Phase 1b: Determining the Optimal Value of Diabetes Risk Score for Identifying People With Undiagnosed Diabetes and Prediabetes

A total of 972 people left their contact information for the project team and were included in the invitation in this phase. Only 210 participants (21.6%) agreed to attend the health assessment. Of these, we excluded 22 people who had type 1 or type 2 diabetes from the study, so eventually we included only 188 app users in the analysis.

**Table 1:** Demographics of the users of the Diabetes Risk Score mobile app.

Variables		Phase 1a (n=4549)	Phase 1b			Phases 2a and 2b (n=127)		
			HbA_1c_^a^ <39 mmol/mol (5.7%), (n=109), n (%)	HbA_1c_ ≥39 mmol/mol (5.7%), (n=79), n (%)	*P* value	DRS^b^<9, n (%)	DRS≥9, n (%)	*P* value
**Sex**								
	Male	2738 (60.19)	66 (60.6)	50 (63)	.62	82 (64.6)	122 (61.3)	.56
	Female	1811 (39.81)	43 (39.5)	29 (37)		45 (35.4)	77 (38.7)	
**Age (years)**								
	≤44	1005 (22.09)	14 (12.8)	4 (5)	.43	23 (18.1)	4 (2.0)	<.001
	45-54	610 (13.4)	45 (41.3)	31 (39)		15 (11.8)	26 (13.1)	
	55-64	1328 (29.19)	41 (37.6)	36 (46)		35 (27.6)	79 (39.7)	
	≥65	1606 (35.30)	9 (8.3)	8 (10)		54 (42.5)	90 (45.2)	
**Educational level**								
	Primary or below	914 (20.1)	2 (1.8)	4 (5)	.76	17 (13.4)	29 (14.6)	.31
	Secondary	1724 (37.90)	63 (57.8)	49 (62)		60 (47.2)	76 (38.2)	
	Tertiary or higher	1911 (42.01)	44 (40.4)	26 (33)		46 (36.2)	85 (42.7)	

^a^HbA_1c_: glycated hemoglobin.

^b^DRS: diabetes risk score, based on the Finnish Diabetes Risk Score.

Most of these participants were aged between 45 and 64 years (153/188, 81.4%) and 116 (61.7%) were male. These app users had a relatively higher educational level than the general public, with 182 (96.8%) reporting secondary school or higher qualifications [27].

#### Results of HbA_1c_ Tests

Among the 188 users who participated in blood tests, 79 (42.0%) had HbA_1c_ of 39 mmol/mol or greater (ie, 5.7%). Thus, we considered them to have UDPD. Of these 79 participants, 14 (17.7%) had an HbA_1c_ even higher than 48 mmol/mol (ie, 6.5%), and we considered them to have undiagnosed diabetes. There were no significant differences in age, sex, and educational level between app users with UDPD and those with normal HbA_1c_ level (Table 1).

#### Optimal Cutoff Value for Diabetes Risk Score in the Chinese Population

**Figure 1:**
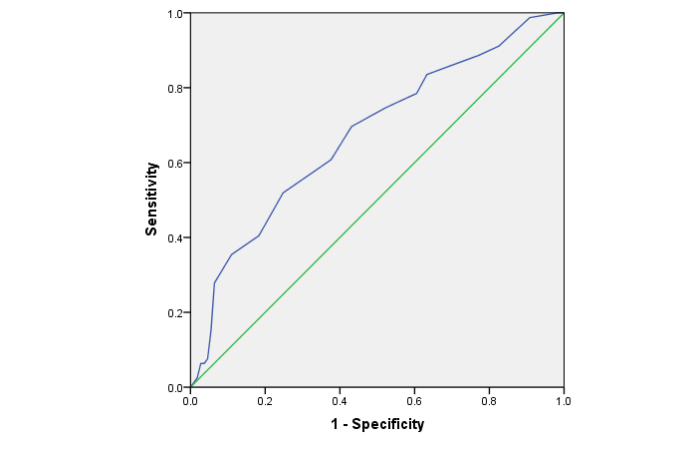
Receiver operating characteristic (ROC) curve analysis of the performance of the Finnish Diabetes Risk Score (FINDRISC) in identifying undiagnosed diabetes and prediabetes. Diagonal segments are produced by ties. The area under the ROC was 0.67 (95% CI 0.60-0.74). When the FINDRISC cutoff value was >8, its sensitivity was 0.70 (95% CI 0.58-0.80) and specificity was 0.57 (95% CI 0.47-0.66).

Table 2 shows the sensitivity and specificity of various FINDRISCs in relation to UDPD. The sensitivity and specificity of a FINDRISC greater than 8 were 0.70 (95% CI 0.58-0.80) and 0.57 (95% CI 0.47-0.66), respectively, with positive predictive value of 0.54 (95% CI 0.44-0.64) and negative predictive value of 0.72 (95% CI 0.61-0.81). A FINDRISC of greater than 8 also had the greatest Youden index of 1.27. When using a FINDRISC greater than 9, specificity increased to 0.62 (95% CI 0.53 to 0.72), sensitivity decreased to 0.61 (95% CI 0.49 to 0.72), and the Youden index decreased to 1.23. The AUC of a FINDRISC greater than 8 was 0.67 (95% CI 0.60-0.74; *P*<.001; Figure 1).

### Phase 2a: Estimating the Chance of Developing Diabetes Within 2 Years

In phases 2a and 2b, 326 app users replied to the invitation emails and completed the online survey. Nearly half of the app users were aged 65 or over (144/326, 44.2%), and only 27 (8.3%) were aged 44 years old or younger. Most of these app users were male (204/326, 62.6%). Regarding their educational level, 136 (41.7%) of these app users had secondary qualifications and 131 (40.2%) had tertiary qualifications or higher. Among these, we considered 199 app users (61.0%) to be in the high-risk group when a diabetes risk score of 9 or higher was applied. People in the high-risk group were older than those in the low-risk group (*P*<.001; Table 1). There were no significant differences in sex and educational level between the 2 groups.

The mean follow-up time of the app users was 22.66 (SD 5.83) months. After a nearly 2-year follow-up, 15 participants had developed diabetes. The mean time of diagnosis of diabetes during follow-up was 12.93 (SD 8.28) months, ranging from 1 to 25 months. The incidence rate of diabetes was 25.56 per 1000 person-years. For the high-risk group, the incidence rate of diabetes was 36.50 per 1000 person-years. For the low-risk group, the incidence rate of diabetes was 8.59 per 1000 person-years. Fisher exact test showed that the association between the risk groups and diabetic incidence was marginally insignificant (*P*=.06).

Table 3 shows the association between the risk groups and diabetes incidence. In model 1 (the unadjusted logistic regression model), there was a marginally insignificant association between the risk groups and diabetes incidence (OR 4.37, 95% CI 0.97 to 19.69; *P*=.06). However, in model 2, after adjustment for sex and educational level, app users in the high-risk group had a significantly higher chance of developing diabetes (OR 4.59, 95% CI 1.01-20.81; *P*=.048) than did the low-risk group. The Hosmer-Lemeshow test gave a *P* value of .91, which implied that the regression model had an acceptable fitness.

### Phase 2b: Lifestyle Changes Among High-Risk App Users

App users who were informed that they had a higher risk of developing diabetes improved their daily intake of vegetables (baseline: mean 0.76, SD 0.43; follow-up: mean 0.93, SD 0.26; *t*_81_=–3.77, *P*<.001) and daily physical activities (baseline: mean 0.40, SD 0.49; follow-up: mean 0.54, SD 0.50; *t*_81_=–2.08, *P*=.04). However, we found no significant change in their smoking status (baseline: mean 0.15, SD 0.36; follow-up: mean 0.09, SD 0.28; *t*_81_=1.92, *P*=.06) or their alcohol consumption (baseline: mean 0.07, SD 0.26; follow-up: mean 0.04, SD 0.19; *t*_81_=1.35, *P*=18).

**Table 2:** Characteristics of the Finnish Diabetes Risk Score (FINDRISC) using different cutoff values to predict undiagnosed diabetes and prediabetes (glycated hemoglobin ≥39 mmol/mol, or 5.7%).

FINDRISC cutoff values	Sensitivity (95% CI)	Specificity (95% CI)	Positive predictive value (95% CI)	Negative predictive value (95% CI)
>6	0.78 (0.68-0.87)	0.39 (0.30-0.49)	0.48 (0.40-0.57)	0.72 (0.59-0.83)
>7	0.75 (0.64-0.84)	0.48 (0.38-0.58)	0.51 (0.41-0.60)	0.72 (0.60-0.82)
>8^a^	0.70 (0.58-0.80)	0.57 (0.47-0.66)	0.54 (0.44-0.64)	0.72 (0.61-0.81)
>9	0.61 (0.49-0.72)	0.62 (0.53-0.72)	0.54 (0.43-0.65)	0.69 (0.59-0.78)
>10	0.52 (0.40-0.63)	0.75 (0.66-0.83)	0.60 (0.48-0.72)	0.68 (0.59-0.77)
>11	0.41 (0.30-0.52)	0.82 (0.73-0.88)	0.62 (0.47-0.75)	0.65 (0.57-0.73)

^a^FINDRISC >8 was the optimal value.

**Table 3:** Logistic regression model of diabetes incidence between the high-risk app users and low-risk app users.

Covariates		Model 1 (unadjusted)			Model 2 (adjusted)		
		Odds ratio	95% CI	*P* value	Odds ratio	95% CI	*P* value
**Diabetes risk group**							
	High	4.37	0.97-19.69	.06	4.59	1.01-20.81	.05
	Low (reference)	1	–	–	1	–	–
**Sex**							
	Male	–	–	–	1.21	0.38-3.88	.74
	Female (reference)	–	–	–	1	–	–
**Educational level**							
	Primary or below	–	–	–	1.33	0.42-4.31	.63
	Secondary	–	–	–	0.96	0.19-5.00	.96
	Tertiary or higher (reference)	–	–	–	1	–	–

## Discussion

### Principal Findings

This study examined the development of a mobile app for identifying people with UDPD and its use over a period of 2 years. Both Android and iPhone users found this app accessible, and more than 13,000 people had downloaded and used this app. Although we included only app users in Hong Kong in the analysis, we noted that many downloads were from countries in Asia, the United States, and Europe. This showed the potential for expanding the use of this mobile app to people who can read and understand Chinese around the world. To the best of our knowledge, this app was the first DRS app targeted at a Chinese population for diabetes risk self-assessment in 2014. The American Diabetes Association recommends screening for diabetes at 3-year intervals after the age of 45 years, particularly for those who are overweight (whose BMI is ≥25 kg/m^2^) [28]. However, not many people comply with this recommendation [29]. As many people are reluctant to go for blood tests, this DRS mobile app provides the general public a means for self-assessment before consulting doctors.

This DRS app, adopting FINDRISC as the key measure for estimating diabetes risk, has been shown to be a reliable tool, although it cannot replace diagnostic investigations such as the oral glucose tolerance test, HbA_1c_ level, and clinical judgment. Validated with HbA_1c_ measures, this DRS app performed well in detecting people with UDPD. The ROC curve analysis suggested that a FINDRISC greater than 8 had sufficient properties for identifying persons with UDPD. We considered other values such as FINDRISC greater than 9; however, the sensitivity of this score decreased dramatically to 0.61. Therefore, we would recommend a FINDRISC of 9 as the optimal cutoff point for identifying UDPD in a Chinese population. The sensitivity and specificity of the recommended cutoff points are reasonably good. The new recommended cutoff points in this DRS app could identify nearly 70% of persons at high risk of diabetes. Approximately 1 million of the Hong Kong population were not aware of their UDPD status [18,19]. If they use this app, which has a sensitivity of 70%, at least 0.7 million of them could know their risk earlier and could start preventive actions.

The new recommended optimal cutoff value of FINDRISC in the DRS app was comparable with the cutoff values in other populations (Multimedia Appendix 1), although there was a slight difference [20,30,31]. In the United States, the optimal cutoff value of FINDRISC for identifying undiagnosed diabetes was 11 [31], while in Bulgaria the cutoff point was recommended as 12 (there was no differentiation between sexes) [30], and in Colombia it was 14 (for both men and women) [20]. Our finding was similar to the recommended cutoff point in a study in Isfahan, Iran, but the specificity of the recommended point in our study was much higher than the one shown in the Iranian population [32]. This Iranian study adopted a robust method to evaluate the ability of the FINDRISC to predict diabetes incidence in 7.8 years among 1537 high-risk persons [32]. Thus, this is a good reference for our study. Variations in the recommended cutoff points in different populations may be related to the variation of referenced tests. In these studies, a variety of blood tests (oral glucose tolerance test, fasting blood glucose, and HbA_1c_) were used. Although the cutoff values vary, most of the recommended points are within a reasonable range of scores, as suggested by the original developers of FINDRISC [11]. The findings of our study therefore provide additional information about the cutoff value of FINDRISC in the Chinese population.

In this prospective study, two important pieces of evidence were worth noting. First, this DRS app had not only a concurrent but also a predictive nature (indicating the chance of developing diabetes in the next 2 years). Evidence showed that app users in the high-risk group had a significantly higher chance (4.59 times) of developing diabetes than those in the low-risk group. This finding echoed the findings of previous studies in other populations. In Colombia, the risk of incidence of type 2 diabetes in 1 year among participants with high risk scores was 4.8 times that among the low-risk group [20]. Similar results were also found in another longitudinal study by Janghorbani et al, the risk of diabetes in the second quartile (9≤FINDRISC<13) being 4.3 times that of participants in the lowest quartile (FINDRISC<9) [32]. These results showed that FINDRISC in the mobile app was a useful tool for predicting the incidence of diabetes in the Chinese population.

Second, the app encouraged people to change their lifestyle. App users who were informed of having a high risk of developing diabetes by the DRS app significantly improved their daily intake of vegetables and did more physical activities in the follow-up period. This showed that the DRS app was a practical tool in health promotion for the general public. App users seemed to be more cautious about their lifestyles and started to develop healthier habits that could protect them from serious health problems such as diabetes [33].

Some researchers have developed different sets of diabetes risk score models for the Chinese population in recent years. Tian et al developed the Dagang dysglycemia risk score model to identify UDPD for the oil field working-age population [34]. Another risk score model for detecting type 2 diabetes for a rural adult Chinese population was developed by Zhang and colleagues [35]. Although these two risk models have high AUCs (0.791 and 0.766, respectively), both models consist of invasive items (such as blood taking) and therefore are not recommended for use in mobile apps. A simpler and noninvasive diabetes risk score was developed based on age, waist circumference, and family history of diabetes for undiagnosed diabetes [36]. Nonetheless, its specificities were rather low (0.211 in men and 0.436 in women). Considering the availability of diabetes risk score models, FINDRISC seemed to be an appropriate measure to adopt in a mobile app.

### Limitations

This study has some limitations that need to be addressed. First, we used only the HbA_1c_ test as the diagnostic standard to identify people with UDPD; however, the 75-g oral glucose tolerance test is the reference standard for diagnosing diabetes. We considered HbA_1c_ because it was more convenient and time saving than the oral glucose tolerance test. Second, the BMI and waist circumference cutoff values used in the calculation of FINDRISC are for white populations [15]. These cutoff values might not be the optimal values for Asian populations, which might have affected the sensitivity of the FINDRISC model. Therefore, future studies could revise the BMI and waist circumference cutoff values to ones that are optimal for Asian people. Third, diabetes incidence should be interpreted with caution. We determined the incidence rate of diabetes based on self-reported diabetes at follow-up assessment. Participants with undiagnosed diabetes might have reported themselves as nondiabetic, or participants might have had a diabetes diagnosis much earlier than the follow-up assessment time, and this may have caused detection bias and interval-censored bias. We could not directly communicate with the respondents of the online survey or perform body measurements or blood tests after the online survey. Future studies could use blood samples to validate the incidence of diabetes.

### Implications for Future Research

First, future studies could explore the existence of other predictors such as dietary sodium, beverage, and fat intake to improve the predictive validity of FINDRISC in the Chinese population. Predictors of diabetes have been used to modify FINDRISC. Studies in Germany and the Philippines modified and simplified FINDRISC for their populations [13,37]. Second, the determination of cutoff values of FINDRISC specific for various sociodemographic groups could make the classification of UDPD more accurate for different groups of people. Many other FINDRISC validation studies have analyzed subgroups. Studies in the United States, Colombia, and other countries set up cutoff scores for various groups according to sex, age, or ethnicity [20,31]. The results of our study indicated that educational level was one of the covariates of risk score groups and diabetes. Therefore, future studies involving a larger population sample and focusing on educational level are required to identify the FINDRISC cutoff scores in different sociodemographic groups in the Chinese population.

### Conclusion

This DRS app was a reliable tool for identifying persons who had UDPD. The odds of developing diabetes were much higher among the high-risk app users than among the low-risk users, evidencing the predictive power of the app in diabetes incidence. The app can also encourage high-risk app users to modify their lifestyle for the sake of reducing their progression from prediabetes to diabetes. This is an illustration of the use of a mobile app in health promotion and disease prevention.

## Appendix

Multimedia Appendix 1 Finnish Diabetes Risk Score (FINDRISC) cutoff values for the detection of undiagnosed diabetes, prediabetes, and hyperglycemia in various populations.

## Abbreviations

AUC: area under the curve
BMI: body mass index
DRS app: Diabetes Risk Score app
FINDRISC: Finnish Diabetes Risk Score
HbA_1c_: glycated hemoglobin
ROC: receiver operating characteristic
UDPD: undiagnosed diabetes and prediabetes
