# Supplementary material for: A Mobile App for Identifying Individuals With Undiagnosed Diabetes and Prediabetes and for Promoting Behavior Change: 2-Year Prospective Study
Source: JMIR Mhealth Uhealth. 2018 May 24;6(5):e10662. doi: 10.2196/10662 (PMC5992453; doi:10.2196/10662)
Supplement: Multimedia Appendix 1 [file mhealth_v6i5e10662_app1.pdf]

Table 4. Different Finnish Diabetes Risk Score (FINDRISC) cut-off values for the detection of undiagnosed diabetes, pre-diabetes and hyperglycemia

	Population	Measurement for diabetes	Optimal cut-off value for undiagnosed diabetes	Sensitivity<br>Specificity	ROC	Optimal cut-off value for prediabetes	Sensitivity<br>Specificity	ROC	Optimal cut-off value for hyperglycemia	Sensitivity<br>Specificity	ROC
Zhang et al., 2016	Non-Hispanic White	HbA1c<br>FPG<br>2-h OGTT,<br>(ADA, 2013)	11	74.93%<br>NM	0.76	10	NM<br>NM	0.68			
	Non-Hispanic Black		12	71.25%<br>NM	0.76	10	62.42%<br>NM	0.67			
	Hispanics		11	65.98%<br>NM	0.72	10	55.88%<br>NM	0.65			
	The whole US population		11<br>Women 12<br>Men 10	72.17%;<br>68.60%<br>74.68%;<br>62.74%	0.78<br>0.74	10<br>Women 10<br>Men 9	68.72%<br>60.89%<br>60.94%<br>62.43%	0.70<br>0.66			
Vandersmissen et al., 2015	Employees	Fasting glycemia (WHO, 2006)							12	100%<br>84.1%	NM
Tankova et al., 2011	Bulgarian	OGTT<br>FPG and 2–h plasma glucose (WHO, 2006)	12	78%<br>62%	0.71				10	84%<br>61%	0.70
Gomez-Arbelaez et	Colombia	FPG	Women 14	71.4%	0.72						

al., 2015		OGTT<br>HbA1c<br>(ADA, 2014)	Men 14	NM<br>66.7%<br>NM	0.75					
Hellgren et al., 2012	Swedish	FPG<br>2-h OGTT<br>(WHO, 1998)						15	NM<br>NM	NM
Janghorbani et al., 2013	Isfahan	75-g 2-h OGTT<br>FPG<br>HbA1c	13	83.6%<br>53.9%	0.75					
García-Alcalá et al., 2012	Mexico	Fasting glucose and 2-hour glucose (ADA)						15	NM<br>NM	NM
Ku et al., 2013	Philippines	Fasting blood glucose, casual blood glucose and 2-h OGTT (ADA, 2008)	FINDRISC<br>ModFINDRISC<br>SimpFINDRISC	NM	0.74<br>0.74<br>0.75	NM	0.56<br>0.57<br>0.56		NM	0.663<br>0.669<br>0.639
Makrilakis et al., 2011	Greek	FPG and/or 2-h plasma glucose	15	81.1%<br>59.8%	0.72			15	67.7%<br>67.2%	0.716

NM: Not Mentioned ADA: American Diabetes Association WHO: World Health Organization
FPG: Fasting plasma glucose OGTT: Oral Glucose Tolerance Test HbA1c: Glycated hemoglobin
